# Protein Requirements and Recommendations for Older People: A Review

**DOI:** 10.3390/nu7085311

**Published:** 2015-08-14

**Authors:** Caryl Nowson, Stella O’Connell

**Affiliations:** 1 School of Exercise and Nutrition Sciences, Deakin University, Locked Bag 20000, Waurn Ponds, Geelong 3220, VIC, Australia; 2 School of Exercise and Nutrition Sciences, Deakin University, 221 Burwood Highway, Burwood, Melbourne 3125, VIC, Australia; E-Mail: stella.oconnell@deakin.edu.au

**Keywords:** protein requirements, elderly, muscle, function, strength

## Abstract

Declines in skeletal muscle mass and strength are major contributors to increased mortality, morbidity and reduced quality of life in older people. Recommended Dietary Allowances/Intakes have failed to adequately consider the protein requirements of the elderly with respect to function. The aim of this paper was to review definitions of optimal protein status and the evidence base for optimal dietary protein. Current recommended protein intakes for older people do not account for the compensatory loss of muscle mass that occurs on lower protein intakes. Older people have lower rates of protein synthesis and whole-body proteolysis in response to an anabolic stimulus (food or resistance exercise). Recommendations for the level of adequate dietary intake of protein for older people should be informed by evidence derived from functional outcomes. Randomized controlled trials report a clear benefit of increased dietary protein on lean mass gain and leg strength, particularly when combined with resistance exercise. There is good consistent evidence (level III-2 to IV) that consumption of 1.0 to 1.3 g/kg/day dietary protein combined with twice-weekly progressive resistance exercise reduces age-related muscle mass loss. Older people appear to require 1.0 to 1.3 g/kg/day dietary protein to optimize physical function, particularly whilst undertaking resistance exercise recommendations.

## 1. Introduction

Currently, the National Health and Medical Research Council (NH & MRC) Recommended Dietary Intakes for Australians incorporate a small increase in dietary protein requirement for older people over the age of seventy years [1]. This is not a consistent dietary recommendation across all countries, as national dietary protein requirements often do not differ across the adult age range [2,3]. Recently there has been some debate regarding the recommended dietary protein intake for older people and it has been proposed that dietary protein intakes which are considerably higher than the minimum protein requirements may be required for optimal health, particularly in older people [4].

Traditionally, protein requirements have been derived on the basis of supplying sufficient dietary protein to ensure nitrogen balance, based on only a handful of experiments involving older people. At a meeting between representatives of the World Health Organization, the Food and Agriculture Organization of the United Nations and the United Nations University held in 2002, one of the recommendations was the need to review the requirements for those with a high disease burden, including the older population, and it was noted that previous reports had failed to adequately consider the protein needs of older people [5]. Furthermore, the premise that no additional protein allowance is required for older adults as lean body mass (as a percent of body weight) and protein content of the body both decrease with age [2,3] may not be valid. There is now emerging data that optimal health for older people depends on maintaining muscle mass, which requires greater than minimal amounts of dietary protein [6]. It is increasingly being recognized that more robust methods are required for measuring protein requirement and that the results from short-term nitrogen balance studies can provide only limited information on dietary protein requirements of older people [7].

Although there is ongoing debate related to the validity of different methods of estimating dietary protein requirements [6,8], Fukagawa argues that what is needed is studies demonstrating that incremental differences in the amount of dietary protein consumed affect clinically-important outcomes. This would require a new paradigm, with a new set of clinical outcomes being used to define nutritional adequacy with respect to protein [7]. The use of functional outcomes such as physical performance can be measured by the ability to get up out of a chair or gait speed, and these outcomes have been shown to be predictive of mortality and morbidity [9,10,11]. Any assessment of optimal levels of dietary protein would need to be performed in the context of the range of physical activity levels present in the older population, including those who are undertaking the recommended regular weight-bearing activities. Consistent with approaches to reduce chronic disease in younger people, dietary recommendations to reduce health risk and optimize quality of life in the later years should be combined with recommendations for physical activity. Therefore, it is timely to review evidence relating dietary protein intake in older people to physical functional outcomes that impact on quality of life.

The aim was thus to review the current dietary recommendations for dietary protein for older people and the evidence relating dietary intake of protein in older people to functional outcomes that impact on quality of life in the context of different levels of physical activity in the context of the physiological ageing process.

## 2. Results

### 2.1. Physiological Impact of Ageing on Body Composition

The largest single site of protein in the body is skeletal muscle, which makes up about 80% of the cell mass and 30% of whole body protein turnover in lean young adults [12]. Each cell contains protein and that protein exhibits both functional and structural properties. In addition, there is a small amount of body protein which can be used to provide energy in the labile amino acid pools and during starvation.

**Figure 1:**
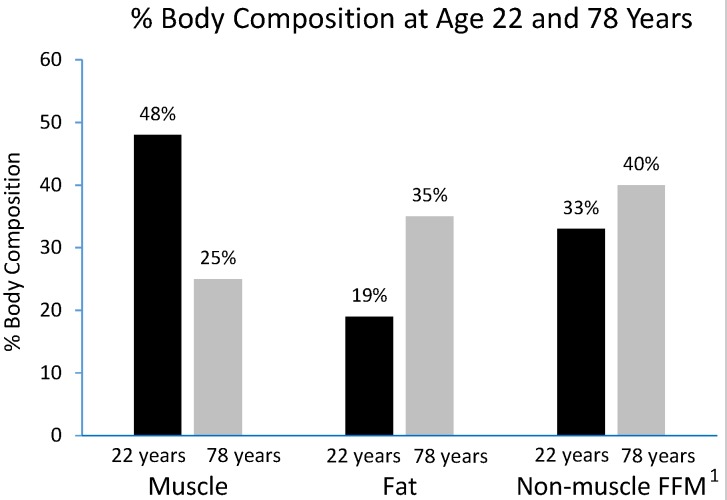
Changes in the relative weights of the different compartments of body composition with ageing. Values are expressed as percentage of total body mass, adapted from Short *et al.*, 2000 [12]. ^1^ Fat-free mass.

Aging is associated with a progressive decline in resting metabolic rate (RMR) at a rate of 1%–2% per decade after 20 years of age [13]. This reduction in RMR is closely linked with the decrease in whole body fat-free mass, which is composed of metabolically-active tissues and organs [14]. Up to 50% of total body weight in young adults is lean muscle mass but this declines with aging to 25% when reaching an age of 75–80 years [12]. The loss of muscle mass is usually coupled with gains in fat mass without much fluctuation in body weight (Figure 1). The greatest loss of muscle mass is seen in the lower limb muscle groups, with the cross-sectional area of the *vastus lateralis* being reduced by as much as 40% between the age of 20 and 80 years [15].

### 2.2. Skeletal Muscle, Bone Health, Falls and Fractures

Muscle weakness predicts falls and subsequent fractures. Muscle weakness is associated with age-related muscle loss which in turn is related to osteoporosis and leads to a life of restricted mobility, loss of independence and reduced life expectancy [16]. Fracture risk is increased when bone density is low, and loss of muscle mass is associated with loss of bone mass. The skeletal and muscular organs are inextricably linked: increasing muscle strength increases bone density. Muscle contractions provide the strongest mechanical forces upon bone, stimulating healthy bone turnover, optimising bone density, bone strength and microarchitecture [16].

Frailty is important because it confers a much greater risk of adverse health outcomes, such as falls, hospitalization, disability, loss of independent living and death [17]. The Fried Frailty Index classifies frailty on the presence of three or more of five components: weight loss, exhaustion, weakness, slowness and low physical activity [17]. The occurrence of frailty increases incrementally with advancing age, and is more common in older women (~10%) than men (~5%), and among those of lower socio-economic status. Between 6% and 25% of free-living individuals aged 65 years and older may be considered frail and this proportion increases to about 25% to 40% in those aged 80 years and above [18].

Physiological reserve is often limited as people age. The progressive restriction of homeostatic reserve that is seen in all organ systems with ageing makes older people more vulnerable to relatively minor pathological conditions, and is thought to underpin the syndrome of frailty. More advanced stages of frailty are associated with muscle weakness and increasing disability. Frailty and age-related accelerated muscle loss (sarcopenia) are closely related and frail older people are by definition sarcopenic [19,20]. Sarcopenia is a complex process involving a range of age-related physiological changes combined with adoption of a sedentary lifestyle and a dietary pattern that is sub-optimal [21]. The two main features of sarcopenia are loss of fast-twitch type II muscle fibres and loss of motor neurons, and prevention of sarcopenia is recognised as a key factor in preventing fracture in older people. Age-associated sarcopenia is likely to lead to frailty, risk of falls, and loss of independence. There is now a defined subclass of sarcopenia that incorporates assessment of physical function: “Sarcopenia with limited mobility” is defined as a person with muscle loss whose walking speed is equal to or less than 1 m/s or who walks less than 400 m during a 6-min walk, and who has a lean appendicular mass corrected for height squared of two standard deviations or more below the mean of healthy persons between 20 and 30 years of age of the same ethnic group, and clinically significant interventions can be defined by walking speed [22].

### 2.3. Energy Expenditure, Energy Intake and Physical Activity

Maintaining adequate physical activity is likely to ameliorate the age-related decrease in resting metabolic rate (RMR), as physical activity aids in preservation of lean mass among highly active older adults [14]. It has been estimated that 75 year-old have total energy expenditure (EE) levels similar to a 7–11 year old despite having greater body mass. This represents a decrease of approximately 2.1 MJ (500 kcal)/day in total EE and therefore in energy requirements [14].

#### 2.3.1. Gender Differences

Throughout the lifecycle, men and women appear to have similar protein turnover rates, once adjusted for body mass index (BMI) and health status. Some studies have indicated that there may be possible gender differences due at least in part to the differences in body composition, not only in lean body mass but also in the amount of body fat. There is some indication that men have higher whole-body leucine oxidation at rest and during aerobic exercise, even after correcting for lean body mass [23].

When energy intake from other macronutrients is marginal or low, protein is utilised by the body as an energy source. This is likely to be more common in older adults who are sedentary and whose appetite is likely to be small. As highlighted in the WHO/FAO/UNA report, protein: energy ratios increase with age, and are highest for females who are sedentary. The protein: energy ratio is highest when energy requirements are lowest, e.g., sedentary elderly large women, such that a sedentary elderly woman who weighed 70 kg would require food with more than twice the protein concentration relative to energy compared with that needed by very young children [5].

#### 2.3.2. Digestibility of Protein

Older adults appear to exhibit a more positive whole-body protein balance after ingestion of rapidly-absorbed protein sources compared to younger people [24]. In young men, a slow-digested dietary protein (casein) induced a greater protein gain than a fast-digested one (whey protein). The mechanisms of this gain also differed according to the protein’s rate of digestion. However, in older subjects, protein gain was greater with whey protein (rapidly-digested protein), and lower with casein (slowly-digested protein). This suggests that a “fast” protein might be more beneficial to limit protein losses in older people, but longer-term studies are required to confirm that this approach reduces body protein losses during aging [24,25]. There is also some recent evidence for greater gains in muscle strength in an elderly population in response to resistance training when this is combined with a 2 × 10 g/day whey protein supplement rather than the same amount of a casein protein supplement [26].

In addition to different absorption and metabolism rates between different food sources, it is clear that ageing is associated with decreased chewing efficiency. For example, minced beef has been shown to be more rapidly-digested than servings of intact beef, resulting in increased amino acid availability and greater postprandial protein retention, although this acute study did not show greater postprandial muscle protein synthesis (MPS) [27].

#### 2.3.3. Dietary Requirement for Protein and Optimal Intake of Protein

There are a number of factors that influence the minimum protein requirement and these include: metabolic demand (with large inter- and intra-individual variation), growth/net tissue deposition, and dietary influences. The provision of sufficient dietary protein will only occur when the demands for *energy* and *all other nutrients* for normal physiological functioning are met. Protein and amino acid metabolism are affected by alterations in micronutrient status and intake along with other factors, as shown below:

- Energy intake—at constant levels of energy expenditure, increased energy intake improves nitrogen balance. This is possibly due to hormonal responses (insulin) which can inhibit proteolysis and oxidation of amino acids. Excess dietary energy over the longer term leads to increased deposition of adipose tissue (with some accompanying lean mass) which increases protein requirements.
- Energy expenditure—an active person expends more energy and consumes more food (including protein), and is therefore less likely to be consuming marginal amounts of protein, as usually there is a minimal or no increase in amino acid and nitrogen requirements due to the physical activity. In contrast, when energy expenditure is low, food consumption falls and any imbalance between amino acid requirements and intake will be more likely to occur.
- Physical activity—can influence amino acid metabolism, e.g., altering the flow of nitrogen from branched-chain amino acids, and training may alter amino acid metabolism.
- Macronutrients—the pattern of consumption of complex and non-digestible carbohydrate affects the colonic microflora and their contribution as an energy source, and faecal nitrogen is increased in those consuming large amounts of non-digestible carbohydrate.
- Micronutrients—dietary inadequacies with respect to B vitamins and zinc may affect the biological value of dietary protein, whereas larger nutrient intakes (supplements) can induce an increased metabolic demand for protein to dispose of excess nutrients.
- Metabolic stress—induced by systemic inflammatory responses, specific immune responses to infections, and other environmental factors, including smoking [28] and large intakes of alcohol [29].

#### 2.3.4. Recommended Dietary Protein Intakes

In contrast to the US and the UK values [2,3], the Australian Recommended Daily Intake (RDIs) for protein distinguish between males and females and recommend a 25% higher intake of dietary protein for those aged 70+ years (Table 1) [30]. In contrast, the US recommends an intake of 0.8 g/day for all adults which does not increase with age but currently remains at the same level as for younger adults. In a recent review (2013), after evaluating the evidence, an international expert panel recommended an average daily intake of 1.0–1.2 g/kg protein per day for those aged 65+ years and even higher intakes for those who are exercising and otherwise active [4].

**Table 1:** Nutrient reference intakes and recommended dietary intakes/allowances and proposed recommended protein intakes for older people (g/kg/day).

	Males ^1^	Males	Males	Females ^1^	Females	Females
Age range (years)	19–50	51–70	70+	19–50	51–70	70+
US EAR	0.66	0.66	0.66	0.66	0.66	0.66
Australian EAR	0.68	0.68	0.86	0.60	0.60	0.75
US RDA	0.8	0.8	0.8	0.8	0.8	0.8
Australian RDI	0.84	0.84	1.07	0.75	0.75	0.94
UK NRI	0.8	0.8	0.8	0.8	0.8	0.8
* General Recommendation (>65 year)		1.1–1.2			1.1–1.2	
* Recommendation with endurance and resistance exercise (>65 year)		1.2			1.2	
* Recommendation for acute and chronic disease (>65 year)		1.2–1.5			1.2–1.5	
* Recommended 25–30 g per meal (>65 year)						

**^1^** Younger age groups included for comparison; EAR, Estimated Average Requirement: the average daily nutrient intake level estimated to meet the requirements of half of the healthy individuals in a group; RDA, Recommended Daily Allowance and RDI, Recommended Dietary Intake: the average daily dietary intake level sufficient to meet the nutrient requirements of nearly all (97%–98%) healthy individuals in a group; NRI, Nutrient Reference Intake; * [4].

There is increasing evidence that the current Recommended Dietary Intakes for older people of around 0.8 g/kg/day are insufficient to optimise retention of muscle mass, strength and function [31,32]. An early study by Campbell *et al.*, found that healthy older subjects were in negative nitrogen balance after consuming the US protein RDA for 10 days [33]. Subsequent to this, a longitudinal study in older men and women aged 55 to 77 years showed that in response to a 3-month diet containing 0.8 g/kg/day protein, there was a physiological adaptation to this low-protein diet evidenced by a reduction in skeletal muscle whilst whole-body leucine metabolism was maintained [34]. These results indicate that a protein intake of 0.8 g/kg/day is not adequate to completely meet the metabolic and physiological needs of virtually all older people. It is clear from these types of studies that the body adapts to a lower protein intake by breaking down lean mass to maintain nitrogen balance, which will ultimately result in progression to sarcopenia, frailty and reduced quality of life in older people.

#### 2.3.5. Implications for Dietary Protein Requirements for Elderly

It is fundamental, as the basis for assessing its adequate intake, to move from a focus on meeting the dietary protein requirement when it is defined by body nitrogen balance studies to defining *optimal intakes* for health and reduction of chronic disease. In addition, any dietary protein recommendations should be combined with clear recommendations relating to physical activity and maintenance of muscle strength and function.

Whilst a dietary protein recommendation that achieved nitrogen balance per se might be the relevant point to set recommendations in young sedentary persons, it is not relevant to older people, since:

- Individuals adapt to minimum protein diets by lowering nitrogen excretion [35], such that there is no relationship between nitrogen balance and musculoskeletal tissue mass.
- Nitrogen balance studies are only conducted for relatively short periods of time (between 10–15 days) which are insufficient to measure the impact on muscle, bone, and connective tissues which may be adversely affected by minimal protein intakes.
- Few nitrogen balance studies have been conducted on older people. In the most recent meta-analysis, only one study with 16 older individuals (68–84 years) was included among the total of 235 individuals [35].
- The level of protein intake to achieve nitrogen balance exhibits a wide range of variation from 50 g/day to 150 g/day.

### 2.4. Evidence that Older People Need More Protein

#### 2.4.1. Basal Conditions

Many studies have indicated that ageing does not alter muscle protein synthesis or skeletal muscle protein breakdown under basal conditions and this is the generally accepted view [36]. However, some studies have demonstrated age-related reduction in basal protein synthesis and differences could be explained by inadequate sample size, pre-study dietary intake, cultural differences or precision of measurement instruments [37]. One of the key factors influencing the variability in results, as pointed out by Henderson *et al.*, is likely to be that weight-maintaining standardised diets were administered for at least 3 days before the experiment in studies finding an age effect, whereas those finding no age effect did not standardise dietary intake going into the study [38]. Additionally, as noted by Koopman *et al.*, pointed out [37], even minor differences in basal muscle protein synthesis and/or breakdown rate (<10%) would be clinically relevant when calculating their impact over one or more decades before sarcopenia becomes evident. This is an area that warrants further research using standardised diets prior to assessment of basal protein synthesis rates in older people.

#### 2.4.2. Response to Anabolic Stimuli

Protein turnover in skeletal muscle tissue is enhanced by consumption of food, particularly ingestion of amino acids and/or protein which strongly stimulates muscle protein synthesis and inhibits protein breakdown, resulting in a positive net protein balance [37]. Older people appear to have lower rates of protein synthesis and lower rates of whole-body proteolysis in response to an anabolic stimulus (consuming food or performing resistance exercise), which is consistent with overall slower tissue remodelling. It is believed that this reduced response to anabolic stimuli represents one of the key factors responsible for the age-related decline in skeletal muscle mass. The mechanisms that underpin the anabolic resistance to protein and/or amino acid consumption and resistance exercise are not well defined [39]. Cuthbertson *et al.*, have demonstrated that older people have an attenuated rise in the activation of key signalling proteins in the mammalian target rapamycin (mTOR) pathway after ingesting 10 g essential amino acids (EAA) [36]. The branched-chain amino acid leucine is a potent activator of the rapamycin (mTOR) nutrient and energy-sensing signalling pathway in skeletal muscle and appears to be the main anabolic signal responsible for the postprandial increase in muscle protein synthesis [40]. One small acute study conducted in healthy elderly people consuming an “adequate” intake of 0.8 g/kg/day demonstrated that leucine supplementation for 2 weeks (4 g/meal; 3 meals/day) improved muscle protein synthesis [41]. In addition to being the most potent stimulus of protein synthesis in skeletal muscle, leucine may also suppress muscle protein breakdown [42].

#### 2.4.3. Optimal Types of Protein

Evidence is emerging that this “anabolic resistance” in older people can be overcome by ingesting protein supplements or foods that are rich in the essential amino acid leucine [43]. It has been found that older people need greater doses of protein (beef) to enhance the rate of postprandial muscle protein synthesis, e.g., ingestion of 113 g of lean beef (220 kcal, 30 g protein, 10 g EAA) increased mixed muscle fractional synthesis rate by approximately 50% in both young and older persons [44]. This increase in protein synthesis was further enhanced by resistance exercise, with myofibrillar protein synthesis increased with 170 g of beef to a greater extent than all other doses at rest and after resistance exercise [45]. However, the evidence indicating the usefulness of leucine supplementation in improving protein anabolism is derived from small acute studies and the impact of consuming leucine (or leucine-rich whey protein supplements) on long-term health needs to be evaluated.

#### 2.4.4. Optimal Distribution of Protein

There is an improved protein synthetic response to intact protein sources such as whey protein, milk and beef. However, if total ingested protein content is low (*i.e.*, EAA content less than ~7 g or protein less than 20 g) or if glucose and amino acids are co-ingested, then the protein synthetic response appears to be impaired in older people [36,46,47]. It appears that ingestion of approximately 25–30 g of protein per meal maximally stimulates muscle protein synthesis in both young and older individuals. Many older people may be consuming only minimal amounts of protein at each meal throughout the day and may need to reach the threshold intake of 25–30 g protein to stimulate protein synthesis. Foods such as meat are not generally consumed alone but as part of a meal and the anabolic response to a mixed meal in older people needs to be evaluated [46]. Within a meal, protein is generally consumed with carbohydrate: many meals provide 15%–30% energy from protein and 60% energy from carbohydrate. A typical meal may contain about 20 g of protein (approximately 100 g of steak or two eggs) and about 40 g of carbohydrate [48]. A recent study has demonstrated that protein synthesis measured after the consumption of dietary protein-derived amino acids following ingestion of a meal-like amount of protein and carbohydrate does not differ between healthy young *vs.* older men [48], and post-prandial plasma glucose and insulin concentrations were significantly higher in the older group. However, the authors do acknowledge that, although not demonstrated in this study, impairments in the post-prandial muscle protein synthetic response are likely to occur in more frail and sedentary elderly populations.

Importantly, it is now clear that ingestion of extra protein beyond 30 g (or ~10 g EAA) in a single meal does not further enhance the stimulation of muscle protein synthesis. In one well-controlled study, despite a threefold increase in protein and energy content, there was no further increase in protein synthesis after ingestion of a 340 g lean beef serving (660 kcal, 90 g protein) in either younger or older people [44]. A dietary plan that includes 25–30 g of high-quality protein per meal (60 g/day) has been proposed to maximize muscle protein synthesis. A 20 g serving of most animal or plant-based proteins contains 5–8 g of essential amino acids. This has been proposed as a strategy particularly relevant to hospitalised or institutionalized elderly who may be severely limited in their ability to perform any physical activity [40].

#### 2.4.5. Timing of Protein Consumption

Another consideration is the optimal timing of protein consumption in relation to exercise. As maximal muscle protein synthesis occurs approximately 60 min after the end of physical exercise, it would be reasonable to predict that ingestion of protein within this time period would result in the best anabolic response; however, there is no definitive evidence to support this premise [49]. In one study, supplementary protein distributed across 2 meals (15 g after breakfast and 15 g protein after lunch), resulting in a total intake of protein of 27 g at breakfast, 32 g at lunch and 29 g at dinner), produced an improvement in physical performance [50]. It should be noted that on average only 23 g extra protein was consumed per day, which is still a very modest increase in total protein intake [51].

A recent assessment of protein intake in older people in the Netherlands indicated that dietary protein intake averaged 1.1 g/kg/day in community-dwelling and 1.0 g/kg/day in frail older people, with protein intakes being particularly low at breakfast: between 8–10 g protein [52]. At lunchtime, community-dwellers consumed an average of 27 g protein whereas the frail population consumed only 18 g protein. It is clear that many are consuming inadequate amounts of protein at breakfast and the frail group were also consuming insufficient protein at lunch (<30 g), less than the amount required for protein synthesis [40]. This low protein intake in the frail population, particularly at breakfast and lunch, could be contributing to their adverse health profile and lower physical activity levels. Therefore, there seems also to be scope to improve the distribution of protein intake throughout the day in older people.

### 2.5. Dietary Protein and Sarcopenia

Prevention of sarcopenia is important for reducing risk of fracture and osteoporosis and it appears that higher levels of dietary protein, particularly when combined with resistance exercise, can effectively reduce fracture risk and osteoporosis. Data from longitudinal studies support the premise that higher dietary intakes of protein reduce age-related muscle loss [53,54,55] and only one failed to find an association with protein intake [56] (Table 2). For example, in a 3-year longitudinal study in 2066 people, those in the highest quintile of protein intake lost approximately 40% less lean mass than did those in the lowest quintile of protein [53]. A study that followed over 300 older people for 10 years (average age 72 years at baseline) [57] found that those on protein intakes in the range 1.20–1.76 g/kg/day had better health outcomes compared to those with protein intake less than the mid-point within the range 0.8–1.2 g/kg/day. The Women’s Health Initiative, with over 24,000 women aged 65 to 79 years followed for 3 years, reported that a 20% increase in protein intake (% kcal) was associated with a 12% lower risk of frailty [58]. One large cross-sectional study has demonstrated that higher protein intakes were positively associated with physical performance [59]. In addition to these epidemiological studies, one protein-supplementation RCT [50] and one community-based RCT in older people [60] demonstrated improvements in physical performance while another hospital-based study in hip fracture patients [61] demonstrated a reduction in medical complications. The study which provided an additional 30 g protein/day for 24 weeks and conducted in a frail community population [50] clearly demonstrated an improvement of more than 1 point for the Short Physical Performance Battery. Overall, these studies indicate that consumption of higher amounts of dietary protein assists in reducing age-related muscle mass and improves health outcomes and physical performance.

#### Protein, Exercise and Prevention of Sarcopenia

The current guidelines for older people call for progressive resistance training to be performed on at least two non-consecutive days of the week [62]. As resistance exercise several times per week is considered to be crucial to maintain muscle strength, maintain muscle mass, and reduce falls and fractures [16], it is important that nutritional interventions are assessed within the context of recommended exercise regimes. It is therefore relevant to assess the outcomes of randomised controlled trials that have addressed the impact on muscle mass and strength of the combined approach of resistance exercise and increased dietary protein, *i.e.*, protein combined with exercise. A meta-analysis of randomised controlled trials that assessed the impact on lean mass, muscle fibre cross-sectional area and/or 1-repetition muscle strength of combined prolonged resistance-type exercise training with increased dietary protein indicated that, for those ≥50 years, an average of 42 g protein on training days increased total lean mass to a degree similar to that in younger people [63]. Most studies used milk or whey supplementation. Overall, dietary protein supplementation during resistance-type exercise training increased FFM by an additional 38% when compared with the placebo. There was no difference between older and younger people with respect to improved lean mass and leg strength, but older people did not experience an improvement in type 1 muscle fibre. Protein supplementation improved FFM gain by 0.48 kg in older people and 0.81 kg in younger people, although there was no statistical difference between the groups. Protein supplementation had a similar effect on improving 1-RM leg press strength in both age-groups (pooled estimate of 14.4 kg in younger people and 13.1 kg in older people). The three studies without energy restriction published after this do not alter the overall conclusion from the meta-analysis, with one finding an improvement in lean mass [64] and the others no improvement [65,66] (Table 1). There may be some limitation in using DEXA to measure body composition in older people, as FFM and muscle protein mass could be overestimated in this group due to water retention and increased lipid content of muscle, but this would not affect muscle fractional synthesis rates.

The effectiveness of exercise in increasing muscle mass and function has been clearly demonstrated even in frail older people [67,68]. Many experts recommend that older people should combine resistance exercise with a protein intake of up to 1.3 g/kg/day [49,69]. One recent RCT has clearly demonstrated that a modest increase in dietary protein (30 g/day/24 weeks) combined with twice-weekly progressive resistance training in a frail elderly population [64] induced an average 1.3 kg increase in lean body mass, although there was no protein effect on strength or physical performance, with both groups experiencing a similar improvement. Obese older adults (mean age 63 years) undertook a weight-loss diet and combined resistance training three times per week with consumption of a protein supplement amounting to an extra intake of 28 g/day, including 21 g immediately after training, or a placebo [70]. In this recent RCT, both groups lost weight but the protein-supplemented group maintained their lean mass whilst the placebo group lost significant muscle. However, both groups improved similarly in muscle strength or function with the extra protein conferring no measureable benefit over placebo treatment. Another study compared the combination of resistance training (3 times/week) with one of two energy-restricted diets (1.3 MJ (300 kcal) energy deficit), both providing 1.3 g/kg/day, over 4 weeks in older men. One diet had an even distribution of protein throughout the day (utilising whey protein drinks) and the other had a skewed intake, with the majority of protein being provided with the evening meal. It was found that there was greater myofibrillar protein synthesis over the day with the balanced protein distribution compared to the skewed distribution under conditions of energy restriction [71]. Although this study utilised whey protein supplements to distribute protein throughout the day and more research is required to determine if these acute observations translate to mixed macronutrient meals and into a long-term functional response, these results lend support to the hypothesis that the combination of RT and a balanced distribution of daily protein in the context of a modest increase in dietary protein could be an effective strategy for fat mass loss during weight reduction without exacerbating sarcopenic muscle loss in older people. It is encouraging that protein treatment has the potential to ameliorate the effects of the weight loss on muscle, since lean mass loss increases risk of sarcopenia in the elderly and its attendant increased risk of morbidity [72].

In contrast, another recent trial in active women 60 years and older used increased red meat consumption to increase protein intake. In this study, all participants undertook progressive resistance training (twice-weekly) and half received additional dietary protein (red meat, consumed at lunch and dinner) which took their total protein intake to ~1.29 g/kg/day, compared to the carbohydrate control diet intake of ~1.15 g/kg/day. The protein/meat group experienced greater gains in total body lean mass (net benefit 0.45 kg) and muscle strength (net benefit 18%) [73]. The results of both these studies confirm that when performing the recommended level of resistance exercise [62], a very modest increase in protein intake which nonetheless results in an intake significantly above the RDI of 1.0 g/kg/day is effective in increasing lean mass.

**Table 2:** Protein intakes and physical function, muscle mass, and strength.

Author & Date Location	Study Type Follow-up Duration	NHMRC Grade # ^1^	Gender, Mean Age	*N*	Mean Protein Intakes and Outcomes
**Cross-sectional and Longitudinal Studies**					
Martin, Aihie Sayer *et al.*, 2011 [59] UK	Cross-sectional	IV	Male, Female 68 year	*N* = 628	*Protein intake ~ 89 g/day, 1.1 g/kg/day in men & 81 g/day, 1.2 g/kg/day in women* **Physical performance** Higher protein intake associated with faster 3 min walk time. Protein intake increase of 1% total energy (*4.9* g/day, *0.07* g/kg/day) associated with 0.037 s increased walking speed (˜1.06%). Significant in women only.
Bartali, Frongillo *et al.*, 2012 [56] Italy	Longitudinal 3-year	III-2	Male, Female 73 year	*N* = 598	*Baseline protein intake 77 g/day.* **Muscle Strength** Baseline protein intake not associated with changes in muscle strength over 3 years. In persons with high levels of inflammatory markers, lower baseline protein intake associated with decline in muscle strength after 3 years.
Vellas, Hunt *et al.*, 1997 [57] USA	Longitudinal 10-year	III-2	Male, Female 72 year	*N* = 304 166 women	*Baseline protein intake in women ~1.0 g/kg/day. Not reported for men* **Change in Physical Health** Baseline protein intakes greater than US RDA associated with fewer health problems during next 10 years in women (not men). Protein intakes 1.2–1.8 (midpoint 1.5) g/kg/day associated with fewer subsequent health problems than <0.8 g/kg/day, including major medical illness/surgery, significant medication, gait and/or balance abnormalities or poor score in activities of daily living assessment or mini mental state examination.
Beasley, LaCroix *et al.*, 2010 [58] USA	Prospective cohort study 3-year	III-2	Female 65–79 year, Median 72 year	*N* = 24,417	*Protein intake 73 g/day, 1.1 g/kg/day (median value; mean not available)* **Frailty** Protein intake inversely associated with risk of incident frailty. 20% increase in protein intake associated with 12% (95% CI: 8%–16%) lower risk of frailty.
Houston, Nicklas *et al.*, 2008 [53] USA	Longitudinal 3-year	III-2	Male, Female 75 year	*N* = 2066	*Baseline protein intake 70 g/day in men, 61 g/day in women, both ~0.9 g/kg/day* **Muscle mass loss** Higher protein intake associated with reduced lean mass loss. After 3 years, Q5 (91 g/day, 1.1 g/kg/day) lost 43% less LM and 39% less aLM than Q1 (57 g/day, 0.7 g/kg/day); *p* for trend <0.01.
Meng, Zhu *et al.*, 2009 [54] Australia	Longitudinal 5-year	III-2	F 75 year	*N* = 862	*Baseline protein intake 81 g/day, 1.2 g/kg/day* **Muscle mass loss** Higher protein intake associated with reduced whole body and aLM loss. T3 (mean 111 g/day, 1.6 g/kg/day) had 5.4%–6.0% higher LM and aLM and upper arm muscle area than T1 (mean 54 g/day, 0.8 g/kg/day).
Scott, Blizzard *et al.*, 2010 [55] Australia	Longitudinal 2.6-year	III-2	Male, Female 63 year	*N* = 740	*Baseline protein intake 88 g/day, ~1.1 g/kg/day* **Muscle mass loss** Higher protein intake (>Australian RDI) associated with higher appendicular lean mass cross-sectionally and follow-up. Protein intake positively associated with muscle mass (aLM) and rate of muscle loss, but not strength. Women: for < 70 year, intake at least 46 g/d (0.8 g/kg/day) and for > 70 years, at least 57 g/day (0.9 g/kg/day), *+3.4%* aLM levels after 2.6 years. Men: for < 70 year men, intake at least 64 g/day (0.8 g/kg/day) and for > 70 year, at least 81 g/day (1.1 g/kg/day), associated with *+3.4% higher* aLM levels after 2.6 years.
**Intervention Studies (Supplement or Diet alone)**					
Tieland, van de Rest *et al.*, 2012 [50] Netherlands	RCT 6 months. Parallel 2-arm. 15 g milk-protein in 250 mL drink or placebo after breakfast and again after lunch.	II	Male, Female 79 year	*N* = 65 Treatment = 34 Placebo = 31	*Baseline protein intake 76 g/day, 1.0 g/kg/day; 30 g/day supplement increased intake to 1.4 g/kg/day* **Physical performance** Protein supplementation significantly improved physical performance; Protein-supplemented *vs.* placebo group: performance score improvement: 12% *vs*. 1% (*p* = 0.02), reduction in time taken to rise from chair: 2.6 s *vs*. 2.3 s (*p* = 0.055), leg extension strength improvement; 19% *vs*. 11%, *p* = 0.059. Leg extension strength tended to increase to a greater extent, but did not affect muscle mass or muscle fiber size.
Kim *et al.*, 2013 [60] Korea	Randomized trial 3 months. Parallel trial, 25 g protein in 2 × 200 mL/day multi-nutrient drink, no placebo.	II	Male, Female 78 year	*N* = 87 Treatment = 43 Control = 44	*Baseline protein intake 36 g/day, ~0.8g/kg/day increased intake to 55 g/day, 1.1 g/kg/day* **Physical performance** Protein supplementation significantly improved physical performance. Protein supplementation improved physical function (PF test of disability) (+5.9%), and maintained Short Physical Performance Battery test score (walking speed, balance tests *etc.*) at 12.5% higher than control. Protein supplementation improved Timed Up-and-Go by 7.2%; controls worsened by 3.4%.
Espaulella, Guyer *et al.*, 2000 [61] Spain	RCT 2 months. Double-blind, 2-arm trial. 20 g protein in 200 mL/day drink or placebo at night.	II	Male, Female 83 year	*N* = 171 Treatment = 85 Placebo = 86	*Dietary protein intake not reported* **Physical performance** Protein supplementation did not improve physical performance more than placebo. **Hospital complication rate** Protein supplementation reduced in-hospital and overall complication rate. Protein supplement group suffered fewer in-hospital complications (odds ratio 1.88 (95% CI 1.01–3.53), *p* = 0.05) and total complications (odds ratio 1.94 (95% CI 1.02–3.7), *p* = 0.04) than placebo.
**Intervention Studies: Diet combined with Exercise**					
Daly *et al*., 2014 [73] Australia	RCT 4 months. Parallel, 2-arm trial. RT 2 times/week plus 2 × 80 g /day lean red meat or 1 serve carbohydrate/day	II	Female 72 year	*N* = 100 Treatment(meat diet) = 53 Control = 47	*Protein intake 1.3 g/kg/day compared with 1.1 g/kg/day* **Muscle mass and Strength** Protein-enriched (meat) group: greater gains in total body lean mass (mean 0.45 kg), leg lean mass (0.22 kg) and muscle strength (18%).
Tieland, Dirks *et al.*, 2012 [64] Netherlands	RCT 6 months. Double-blind, parallel 2-arm trial. RT 2 times /week plus 15 g milk-protein in 250 mL drink or placebo after breakfast and again after lunch	II	Male, Female 78 year	*N* = 62 Treat = 31, Placebo = 31	*Protein intake 77 g/day, 1.0 g/kg/day; with extra 30 g protein/d, increased to 1.3 g/kg/day* **Muscle mass change** Protein supplementation: increased muscle mass more than exercise alone. Supplementary protein group gained mean 1.3 kg lean mass compared to −0.3 kg in exercise-only group (*p* = 0.006). **Physical performance** Protein supplementation: no extra improvement in strength and physical performance.
Anarson *et al*., 2013 [66] Iceland	RCT 3 months. RT 3 times /week plus post-exercise 20 g milk protein in 250 mL drink or control.	II	Male, Female 74 year	*N* = 161 Treatment = 83, Placebo = 78	*Protein intake 79 g/day, 1.0 g/kg/day increased to 1.0 g/kg/day* **Muscle mass change** Protein supplementation immediately after training: no significant effect on increase in lean mass. Protein-supplemented and placebo: gained on average 0.8 kg LM and 0.6 kg aLM **Physical performance** Protein supplementation after training did not improve strength and performance more than exercise alone. Both groups increased quadriceps strength by 55 N and improved Timed-Up and Go test by 0.6 s.
Leenders, *et al.*, 2013 [65] Netherlands	RCT 6 months. RT 3 times /week plus daily 15 g milk protein supplement in 250 mL drink or lower-energy placebo, post-breakfast.	II	Male, Female 70 year	*N* = 53 completed Treatment = 27, Placebo = 26	*Baseline protein intake in women 1.2 g/kg/day, in men 1.1 g/kg/day increased to approx. 1.4 g/kg/day and 1.3 g/kg/day during intervention.* **Muscle mass change** Protein supplementation: no effect on muscle mass beyond exercise alone. Protein-supplemented and placebo: similar increase in leg muscle mass (women, 3% ± 1% *vs.* 4% ± 1%; men, 3% ± 1% *vs*. 3% ± 1%) and in quadriceps cross-sectional area (women, 9% ± 1% v*s*. 9% ± 1%; men, 10% ± 1% *vs*. 9% ± 1%) (NS). **Physical performance** Protein supplementation: no effect on physical strength and performance beyond exercise alone. Protein-supplemented and placebo: similar increases in One-RM strength: 40% ± 3% *vs.* 45% ± 6% (women) and 44% ± 3% *vs.* 41% ± 4% (men), respectively (NS).
Chalé, *et al.*, 2013 [74]	RCT 6 months. RT 3 times /week, plus daily 2 × 20 g milk protein supplement in powder form or iso-caloric control powder, post-breakfast and post-dinner.	II	Male, Female 77 year	*N* = 80 Treatment = 42, Placebo = 38	*Baseline protein intake approx. 71 g/day, 1.0 g/kg/day in both, changed to 1.2 g/kg/day in treatment and 0.9 g/kg/day in placebo groups* **Muscle mass change** Lean mass increased 1.3% and 0.6% in treatment and placebo groups respectively but not significant. **Physical performance** No difference in muscle strength (1RM) between groups. Greater increase in peak power for knee extensors in protein group. Otherwise, trend to greater improvement in all outcomes in protein group but NS.
Kukuljan S *et al.*, 2009 [75]	RCT 18 months. RT + 2 × 200 mL fortified milk, am and pm (afternoon or evening as desired), or RT only for control	II	Male 60.5 year	*N* = 87 completed Treatment = 43, Control = 44	*Baseline protein intake 1.26 g/kg/day for treatment and 1.32 g/kg/day for control groups, increased by 12 mo to 1.47 and 1.40 g/kg/day (p < 0.01 and p < 0.05), respectively.* **Muscle mass change** Total body lean mass increased by 1.0 kg and 0.7 kg in treatment and placebo groups respectively, but no difference between groups. **Physical performance** No significant difference between groups in functional tests.

^1^ Australian NHMRC Levels of evidence: II Evidence obtained from at least one properly-designed randomised controlled trial; III-2 Evidence obtained from comparative studies (including systematic reviews of such studies) with concurrent controls and allocation not randomised, cohort studies, case-control studies, or interrupted time series with a control group; IV Evidence obtained from case series, either post-test or pre-test/post-test [76] g/day: grams per day; g/kg/day: grams per kilogram body weight per day; RDA: recommended dietary allowance; RDI: recommended dietary intake; LM: lean mass; (a)LM: (appendicular) LM; RT: resistance training. One (1) RM: One (1) repetition maximum; Q: quartile/quintile, T: tertile. Q1/T1 are lowest protein intakes; T3/Q4/Q5 are highest protein intakes (as appropriate). Mean of tertile/quartile/quintile is stated if provided by reference or else the calculated midpoint of upper and lower values in range is shown. CI: confidence interval. ˜: approximately; NS: non-significant.

Evans *et al.*, in their recent review of the studies by Tieland *et al.* [32,52,64], emphasise that these new trials confirm indications from previous work that older persons require greater dietary protein intakes: between 1.0 and 1.5 g/kg/day. This is specifically required for an optimal increase in muscle mass and presumably long-term physical performance when undertaking regular resistance exercise. As 30 g/day of supplementary protein (twice a day as a component of two meals) does not result in a reduction in habitual energy intake and is not accompanied by any negative health outcomes such as an adverse effect on renal function, they recommend this approach for delivery of a modest increase in protein intake as a key nutritional strategy to improve physical performance and attenuate the progression to sarcopenia and frailty [32]. Although the study by Tieland *et al.* [64] looking at effects of protein-supplementation combined with twice-weekly progressive resistance training failed to show an incremental effect of protein on physical performance, data from the meta-analysis of randomised controlled trials assessing the combination of additional protein with resistance exercise has found a clear improvement in muscle strength [63]. Therefore, the optimistic assertion by the authors that an “increase in skeletal muscle mass in the protein as opposed to the placebo-supplemented group will likely allow a further increase in muscle strength and performance as time progresses” [64] does seem reasonable, despite the fact that there is only a weak relationship between muscle mass and strength in this frail population [77].

Although changes in muscle mass have been shown to be poor predictors of clinical outcomes, they are strongly associated with strength, which in turn is strongly associated with mortality, “demonstrating that muscle strength as a marker of muscle quality is more important than quantity in estimating mortality risk” [78]. Although intervention studies which result in changes in muscle mass do not show a clear direct relationship with muscle strength and physical performance [77,79], these studies have not necessarily been powered to evaluate functional end points in healthy elderly persons [79]. A recent meta-analysis of nine RCTs (462 subjects; varying protocols) which assessed whether protein supplementation could optimize the effects of resistance training on muscle mass and strength in an aged population noted that the variation regarding supplementation protocols, protein sources, and amounts and timing of ingestion limited the results [80]. The authors, however, concluded that combining protein supplementation with resistance training is effective for eliciting gains in fat-free mass among older adults, but does not seem to increase muscle mass or strength, although two trials found generally consistent greater improvements in functional tests in protein-treated groups [64,74]. However, it is likely that larger trials that have demonstrated differences in chronic protein intake spanning the range from inadequate intakes to optimal intakes (<1 g/kg/day to ≥1.2 g/kg/day) over a period of months are required to demonstrate statistical significant functional benefits.

It should be acknowledged that the optimal level of daily protein intake is not yet clear and for individuals will depend on a range of factors, including amount and type of physical activity and exercise undertaken, distribution pattern of dietary protein throughout the day, protein source, *etc*. It is clear that data from studies of longer duration is required and that more attention needs to be paid to the quantity and quality of protein ingested with each meal [81]. It may be that frailer individuals have a greater response to such interventions in terms of physical performance, as they are more likely to be consuming low intakes of protein to begin with (<30 g protein per meal) [52]. It is also likely that with our current eating habits in Australia and other developed countries, many older people are consuming only small amounts of protein earlier in the day at breakfast and/or at lunch, and only consume a meaningful amount of protein (>25 g) at their evening meal.

### 2.6. Future Studies

The positive findings of the three relevant studies to date [50,64,73] provide strong support for conducting further longer-term studies. They raise the possibility that increased dietary protein may prevent mobility disability independently of muscle mass improvement [82] and may act on some other physiological process, possibly related to neural activation of skeletal muscle. Larger randomized clinical trials are needed to establish the optimal forms and timing of dietary protein, the dose ingested, and/or the type and timing of exercise. Any protein supplementation studies need to ensure an adequate energy intake to meet energy requirements, since supplementing protein without an adequate supply of energy will not result in increased protein synthesis as the protein will instead be metabolised as an energy source. An additional factor, often overlooked, is that any intervention found to be beneficial needs to be achievable and acceptable to older people in the long-term, able to be incorporated into a dietary pattern that meets all their dietary requirements whilst enhancing their enjoyment in life. This is why food- and meal-based strategies, rather than supplemental drinks, would be recommended as the initial approach to optimising protein intake. Additionally, it is important that these studies are replicated in the range of elderly population groups and that interventions be evaluated in the long term for sustainability. As already emphasised [83], dietary protein intake and physical activity are the key modifiable means of stimulating muscle protein anabolism. However, there is only a tenuous link between highly-controlled, acute, mechanistic studies and longer-duration, outcome-focused trials. The trials by Daly *et al*., and Tieland *et al.* [64,73] have clearly demonstrated that making the translational leap from a successful acute, mechanistic result to longer-term improvement in outcomes such as muscle mass and function is possible. The final leap is again to ensure that any lifestyle approach is sustainable in the long-term and meets the dietary requirements for all nutrients, whilst enhancing the enjoyment of life in individuals.

## 3. Conclusions

There is new evidence linking protein intake with sarcopenia and physical function, and recent metabolic and epidemiological studies indicate that the current Recommended Dietary Intakes for protein appear to be inadequate for maintenance of physical function and optimal health in older adults. The current body of evidence indicates that a dietary protein intake of at least 1.2 g/kg/day is required to maintain optimal muscle function in older people. Additional studies are required to establish the optimal amount and timing of protein intake for older people [31] within dietary patterns that can be readily achieved by older people living in the community and residential aged care.
